# Accuracy of epilepsy screening tools in community and primary care settings across countries in Sub-Saharan Africa: systematic review and meta-analysis protocol

**DOI:** 10.1136/bmjopen-2026-116684

**Published:** 2026-05-07

**Authors:** Emmanuel Kwame Darkwa, J Helen Cross, Patrick Adjei, Charles R Newton, Arjune Sen, Albert Akpalu, Josemir W Sander, Anthony Danso-Appiah

**Affiliations:** 1 Dodowa Health Research Centre, Research and Development Division, Ghana Health Service, Accra, Ghana; 2 Department of Epidemiology and Disease Control, School of Public Health, University of Ghana, Accra, Ghana; 3 Institute of Child Health, UCL NIHR BRC Great Ormond Street, London, UK; 4 Department of Medicine, University of Ghana Medical School, Accra, Ghana; 5 Department of Medicine, Korle Bu Teaching Hospital, Accra, Ghana; 6 Oxford Epilepsy Research Group, Nuffield Department of Clinical Neurosciences, John Radcliffe Hospital, Oxford, UK; 7 Department of Psychiatry, Oxford University, Oxford, UK; 8 UCL Queen Square Institute of Neurology, London, UK; 9 Chalfont Centre for Epilepsy, Chalfont St Peter, UK; 10 Stichting Epilepsie Instellingen Nederland (SEIN), Heemstede, Netherlands; 11 Department of Neurology, West China Hospital, Sichuan University, Chengdu, China; 12 Centre for Evidence Synthesis and Policy, University of Ghana, Accra, Ghana; 13 Africa Communities of Evidence Synthesis and Translation, Accra, Ghana

**Keywords:** Epilepsy, Sensitivity and Specificity, Meta-Analysis, Seizures

## Abstract

**ABSTRACT:**

**Introduction:**

Circumstantial evidence suggests that a high proportion of cases of epilepsy in countries across sub-Saharan Africa (SSA) remain undiagnosed. The magnitude of the burden is unknown. Screening tools offer promise for early detection and prevalence estimation that will enable evidence-informed management of epilepsy in SSA. This review will systematically assess the accuracy and reliability of screening tools for detecting epilepsy in communities and primary care settings in SSA.

**Methods and analysis:**

Relevant databases, non-database sources and grey literature will be searched for studies on epilepsy screening tools. PubMed, LILACS, CINAHL, PsycINFO and Google Scholar, from inception to 31 May 2026, will be searched for studies on screening tools (questionnaires) administered by non-expert physicians to populations or hospital/clinic-based cohorts with no language restrictions. The following search terms will be used: screening tool, screening questionnaire, screening test, screening instrument, diagnostic tool, diagnostic accuracy, epilepsy, sensitivity, specificity, true positive, false positive, true negative and false negative and SSA. All countries in SSA will be included as search terms. Cochrane databases, African Journals Online, African Index Medicus, HINARI and Preprint and Thesis repositories will also be searched. Reference lists of potentially relevant studies will be reviewed, and experts will be contacted to identify additional studies missed in our searches. Study selection (using a pretested study selection flow chart), data extraction (using a validated data extraction form) and risk-of-bias assessment (using the revised Quality Assessment of Diagnostic Accuracy Studies-2) will be performed independently by at least two reviewers, and any discrepancies will be resolved through discussion. The pooled sensitivity, specificity and diagnostic odds ratio (DOR) will be estimated from 2-by-2 tables of true positives, false positives, true negatives and false negatives. Possible causes of heterogeneity between studies will be assessed through pre-specified subgroup analyses. A meta-analysis will be conducted using a bivariate random-effects model to summarise sensitivity, specificity and DOR per patient. A summary receiver operating characteristic curve will be plotted to determine the overall diagnostic performance of the index tests. Sensitivity analyses will be conducted to test the robustness of pooled estimates of screening accuracy, and all estimates will be presented with their 95% CIs.

**Ethics and dissemination:**

This study will synthesise empirical evidence from publicly available published and unpublished studies, and hence no ethical approval is required. An eligible study with serious ethical issues will be excluded from the analysis and the reasons for exclusion will be documented. The review findings will be shared with all relevant stakeholders, including healthcare providers, patient advocate groups, agencies involved in implementing epilepsy care and policies, civil society, social services providers and researchers. The review findings will be shared widely at scientific symposia and conferences, and the final report will be published in a high-impact-factor peer-reviewed journal.

**PROSPERO registration number:**

CRD42024566976.

**STRENGTHS AND LIMITATIONS OF THIS STUDY**

- This systematic review uses rigorous methods, best practices and reporting guidelines to comprehensively synthesise trustworthy context-sensitive evidence on accuracy of screening tools in sub-Saharan Africa.
- Study selection is guided by the population, index test, reference standard and diagnosis of interest framework, and diagnostic performance will be synthesised using bivariate random-effects models, which appropriately account for the correlation between sensitivity and specificity and between-study heterogeneity.
- By employing the Quality Assessment of Diagnostic Accuracy Studies 2 tool to assess risk of bias, the included studies are critically appraised for quality, thereby ensuring the reliability of the review’s conclusions.
- The review explores between-study heterogeneity through subgroup analyses and sensitivity analyses by setting, population and reference standard, enhancing the robustness and applicability of findings across diverse community and primary care contexts in SSA.
- A possible limitation is that some studies may target different age ranges or epilepsy subtypes, which can affect the sensitivity and specificity of screening tools across demographic groups within the population.

## Introduction

 Epilepsy affects over 50 million people globally; however, the burden is disproportionately high in sub-Saharan Africa (SSA)^1 2^ due to an increased incidence of neuroinfections,^3 4^ perinatal complications^5^ and traumatic brain injuries.^5^,^8^ Despite this high burden, the region faces a significant diagnostic and treatment gap driven by limited access to healthcare,^9^ stigma^8^ and a shortage of specialised personnel.^9^,^13^

Inaccuracies in estimating incidence and prevalence may arise from challenges in disease ascertainment and systematic misclassification of epilepsy cases, potentially leading to incorrect diagnoses.^7 14 15^ In regions where epilepsy carries a significant stigma and where cultural beliefs or negative attitudes contribute to concealing symptoms or avoiding diagnosis, there is a risk of underestimating both prevalence and incidence.^11 16 17^ This underscores the importance of understanding cultural factors that affect the accurate reporting of epilepsy cases, ensuring that public health initiatives effectively address these challenges.

Any study that quantifies the number of affected individuals within a substantial population-based cohort must incorporate a validated measurement tool as a fundamental component.^14^ In the context of epilepsy, this typically involves the utilisation of multi-item screening questionnaires.^14^

Community and primary care screening for epilepsy in resource-limited settings commonly use structured questionnaires^18^ of which several have been applied in SSA, including the Kilifi epilepsy questionnaire,^19^ WHO screening questions^20^ and the Zambian five-item questionnaire.^21^ These questionnaires have varying accuracies with reported sensitivities ranging from 60% to >95% and specificities from 50% to >90%.^19 21 22^ For example, a community-based study conducted in rural Kenya reported sensitivity of 48.6% and specificity of 100%,^6^ and another from rural Zambia reported sensitivity of 64% and specificity of 86%.^21^ Screening questionnaires have emerged as a practical and cost-effective tool for identifying individuals with epilepsy, particularly in resource-constrained settings. These tools can be administered by non-specialist healthcare workers or community health workers, facilitating early detection and referral for appropriate medical care. However, no single universally accepted or standardised questionnaire has been validated for identifying epilepsy in the general population.^14^ Instead, various tools with differing levels of validation have been utilised,^22^,^26^ and the diagnostic accuracy of these screening questionnaires in such a diverse population as SSA remains uncertain. Cultural differences, language variations and differing manifestations of epilepsy can impact the effectiveness of these tools, as well as the screening for both convulsive and nonconvulsive epilepsy.

### Rationale for this systematic review

Although the burden of epilepsy and diagnostic challenges in SSA is well established, the diagnostic accuracy of available screening questionnaires has not been systematically assessed. Reported sensitivity, specificity and predictive values vary widely, and cultural and linguistic differences further complicate their application. Assessing the sensitivity, specificity and positive and negative predictive values of various epilepsy screening questionnaires will help determine their suitability for widespread use in the SSA region. Identifying the most accurate and culturally appropriate tools can aid the development and implementation of targeted screening programmes, ultimately improving early diagnosis and management of epilepsy. Therefore, a systematic review is warranted to synthesise existing evidence, evaluate methodological quality using established frameworks (population, index test, reference standard and diagnosis of interest (PIRD) framework and Quality Assessment of Diagnostic Accuracy Studies 2 (QUADAS-2)) and generate robust estimates of diagnostic performance.

To ensure this systematic review does not duplicate existing work, a comprehensive search was conducted across major research databases, including PubMed, Cochrane Library, Embase and the Database of Abstracts of Reviews of Effects. We did not find any systematic review with SSA focus on this topic. Additionally, registered systematic review protocols were reviewed in platforms such as PROSPERO. This process confirmed that while certain studies focused on the diagnostic accuracy of epilepsy screening tools globally, no systematic review to date has exclusively evaluated the effectiveness of screening tools in SSA.

This review aligns with various United Nations Sustainable Development Goals (SDGs), notably SDG 3, which aims to ensure healthy lives and promote well-being for people of all ages.^27 28^ SDG 3.4 attempts to reduce premature mortality from non-communicable illnesses, such as epilepsy, by promoting early identification and treatment.^28^ SDG 3.8 also promotes universal health coverage,^27^ which includes access to key health services and diagnostics for vulnerable populations in underdeveloped areas such as SSA. Our review findings will enhance epilepsy diagnosis, which contributes to SDG 3 objectives for non-communicable illnesses and equitable healthcare. This review indirectly supports SDG 1,^29^ which aims to eradicate poverty, as epilepsy treatment improves patients’ quality of life and economic productivity, alleviating the socio-economic burden on families and communities. This systematic review will also highlight existing research gaps, guide future studies and contribute to the evidence base required for policymaking and resource allocation. By enhancing early detection and reducing the treatment gap, the review aims to improve health outcomes and reduce the burden of epilepsy in SSA.

This review will answer the following questions: 1) what is the diagnostic accuracy of existing screening questionnaires/tools for identifying epilepsy in individuals in countries across SSA? 2) What factors explain variation in diagnostic accuracy across studies, including differences in screening tools, populations and settings?

### Definitions, terms and concepts

*Epilepsy*: defined as the occurrence of at least two unprovoked (or reflex) seizures occurring >24 hours apart, or one unprovoked (or reflex) seizure with a probability of further seizures similar to the general recurrence risk after two unprovoked seizures (at least 60%) over the subsequent 10 years, or the diagnosis of an epilepsy syndrome.^30^

*Types of epilepsy*:

- *Focal epilepsy* is the type of epilepsy in which seizures originate within networks limited to one hemisphere. They may be discretely localised or more widely distributed.^31^
- *Generalised epilepsy* refers to seizures that originate at some point within, and rapidly engage, bilaterally distributed networks.^31^
- *Convulsive epilepsy* presents with seizures characterised by substantial motor activity. Such activity might be muscle stiffening and/or rhythmic jerking, usually accompanied by loss of consciousness. Common examples include generalised tonic-clonic, focal to bilateral tonic-clonic and tonic seizures.
- *Non-convulsive epilepsy* presents with seizures but without prominent or overt jerking or stiffening, often involving brief impairment of awareness, behaviour or sensation. Common types include absence seizures, focal impaired consciousness seizures and focal preserved consciousness seizures.

*Screening tools* are simple, standardised instruments or questionnaires used to identify individuals in a population who are likely to have a disease, particularly in community or primary care settings. They do not confirm diagnosis but help identify individuals for further clinical assessment.

*Index test* is the test or screening tool under evaluation whose performance is being measured.

*Reference standard* is the best available tool or instrument for determining an individual’s true disease status. In epilepsy studies, this usually involves clinical diagnosis by a neurologist, with or without electroencephalogram.

*A false positive* occurs when an index test incorrectly identifies an individual as having a disease.

*True positive* results occur when the index test correctly identifies an individual who truly has the disease, as confirmed by the reference standard.

*False-negative* results occur when the index test fails to identify an individual who truly has the disease according to the reference standard.

*True-negative* results occur when the index test correctly identifies an individual who does not have the disease, as confirmed by the reference standard.

*Sensitivity* of a screening test measures its ability to correctly identify people who have a disease (true positives), minimising missed cases (false negatives). A highly sensitive test correctly flags most individuals with the condition, making it great for screening to detect as many true cases as possible, but it might produce more false positives. Sensitivity is calculated as true positives / (true positives+false negatives).

*Specificity* of a screening test measures its ability to correctly identify people who do not have a disease, giving a negative result (true negatives). It is the proportion of truly healthy people who are correctly identified as healthy, expressed as true negatives / (true negatives+false positives). High specificity means fewer false positives (people without the disease who test positive).

*Positive predictive value* denotes the probability that individuals who test positive do have the condition.

*Negative predictive value* is the probability that individuals who test negative do not have the condition.

*Accuracy* of a test measures its ability to correctly identify who has a disease (true positives) and who does not (true negatives), using metrics such as sensitivity (true positives) and specificity (true negatives), relative to a trusted reference standard. Key measures include predictive values (the likelihood that a positive/negative result is correct) and likelihood ratios, with results often visualised via receiver operating characteristic (ROC) curves, all of which are influenced by disease prevalence and study quality.

*Likelihood ratios* are the likelihood that a positive test result would occur in an individual with the condition, compared with an individual without the disease.

*A negative likelihood ratio* is the likelihood that a negative test result would occur in an individual with the condition, compared with an individual without the disease.

*Diagnostic OR (DOR)* is the ratio of the odds of a positive test result in individuals with the condition to the odds of a positive test result in individuals without the condition.

*ROC* is a graph that shows a model’s ability to distinguish between two groups (like sick vs healthy) across all possible thresholds, plotting the true-positive rate (sensitivity) against the false-positive rate (1-specificity). It helps in assessing diagnostic test performance and compare models, with the area under the curve (AUC) indicating overall accuracy (a higher AUC is better). Essentially, it visualises the trade-off between correctly identifying positives (sensitivity) and incorrectly flagging negatives as positives (false alarms).

*AUC curve* quantifies a binary classifier’s ability to distinguish between the positive and negative classes, representing the probability that a model ranks a random positive instance higher than a random negative one. An AUC of 1.0 is perfect, and values between 0.7 and 0.9 are generally considered acceptable to excellent for good discrimination, with higher values indicating better performance across all thresholds.

*Characteristics or properties of tools*:

- *Ease of use* is the extent to which a tool can be administered simply and correctly with minimal training, time and technical expertise, without causing burden to users or respondents.
- *Preference* refers to the degree to which users (eg, health workers or respondents) favour a tool over alternatives, based on comfort, familiarity and perceived usefulness.
- *Understandability* is the extent to which the questions and instructions are clear, simple and easily comprehended by both the interviewer and the respondent, particularly in local languages.
- *Appropriateness* refers to the suitability of a tool for the target population, cultural context and study purpose, including whether its content reflects locally relevant presentation of the diseases and experiences.
- *Feasibility* is the practicality of implementing the tool within existing health system constraints, including time, cost, staff availability, infrastructure and workload.
- *Adaptability* is the ability of a tool to be translated, culturally adapted or modified without compromising its validity or reliability, enabling use across different settings or populations.

*Primary care facility* is the first point of contact within the formal health system, providing basic preventive, promotive and curative services to the community.

*Peripheral health facility* refers to lower level, health facilities located close to the communities, often with limited diagnostic capacity and specialised staff.

## Methods

This systematic review protocol has been prepared following guidelines specified in the Cochrane Handbook for diagnostic test accuracy^32^ and reported in line with the Preferred Reporting Items for Systematic Review and Meta-Analysis extension for protocols (PRISMA-P) guidelines^33^ (online supplemental file 1). The specific aspects of the methods have been reported following the formats of earlier published works.^34^,^38^ The full review will be prepared and reported in line with the PRISMA of Diagnostic Test Accuracy (PRISMA-DTA) statement.^39^ The PRISMA flow diagram^39^ (online supplemental file 2) will be used to document the study retrieval process. The full review started on 1 March 2026. Searches and management of the search output were completed on 31 March 2026, and study selection, data extraction and risk-of-bias assessment on 30 April 2026. Data analysis, writing the results and discussion will be completed by 30 June 2026. The review is expected to be completed by 31 July 2026.

### Patient and public involvement

The review questions and outcomes were developed through a collaborative effort between health professionals, policymakers, patients, the public and relevant non-governmental organisations, drawing on their lived experiences, preferences and priorities. This process aligns with the guidelines set out in the Good Reporting of Patient and Public Involvement checklist.^40^

### Types of studies

Studies that assessed the diagnostic accuracy of screening questionnaires to identify people (of all ages and sexes) with epilepsy will be considered. Both telephone-administered and in-person-administered questionnaires used in population-based studies or non-specialist hospital/clinic settings will be eligible for inclusion. To be eligible for inclusion, the study should report true positives, false positives, true negatives and false negatives to enable the calculation of sensitivity, specificity, predictive values and diagnostic accuracy. Studies that reported sensitivity, specificity and predictive values without providing the numbers of true positives, false positives and false negatives will be excluded. In cases where the results of a multi-county study have been lumped together and cannot be disaggregated, such studies will be included and interpreted accordingly.^39^ Studies in which the screening questionnaire was administered by expert health professionals (neurologists, epileptologists or physicians with formal specialised training in epilepsy diagnosis and management) in primary care or community settings will also be included. Reviews, commentaries, case reports, case series and case studies will be excluded, but if any relevant primary studies are identified, they will be eligible for inclusion. Studies that focused primarily on evaluating the effectiveness of epilepsy treatments rather than on the performance of screening tools or the identification of epilepsy cases will be excluded.

### Participants

Individuals of all ages, sexes and ethnic backgrounds residing in a country in SSA, whose epilepsy status has been confirmed or suspected of having epilepsy based on a validated screening tool, will be eligible for inclusion. To be eligible, the tool used for detecting epilepsy and the setting (community, health facility, rural or urban) should have been reported.

### Index test

The index test will include any structured questionnaire or screening instrument designed to identify suspected epilepsy in community-based or primary care settings. Screening questionnaires will be administered both by telephone or in person: these include but are not restricted to the Kilifi Seizure Questionnaire,^41^ which is widely used in community-based epilepsy studies in SSA, particularly in rural settings; the WHO screening questionnaire,^20^ adapted for use in several SSA countries to identify individuals with epilepsy; and the 5-item Zambian screening tool^42^ to detect epilepsy among the general population. A positive or ‘yes’ response to key questions, such as whether the individual has had episodes of loss of consciousness or seizures; episodes of involuntary shaking, jerking movements or convulsions that the individual could not control; episodes of lost consciousness or ‘blackouts’ in which the individual cannot remember the events; or episodes ever fallen to the ground without a reason and bitten tongue or urinary incontinence may indicate the need for further evaluation and confirmation of epilepsy. Tools that were designed for general neurological disorders or mental health conditions without a specific focus on epilepsy will be excluded. Test sensitivity is the proportion of individuals with the target condition who are correctly identified as having it. In contrast, test specificity is the proportion of individuals without the target condition correctly identified as not having it.^43^

### Reference standard

This refers to clinical diagnosis of epilepsy by a neurologist or expert physician that involves a comprehensive evaluation of patient history and physical examination.

### Outcomes

#### Primary outcomes

- *Sensitivity*: the proportion of individuals with epilepsy who are correctly identified as positive by the index test.
- *Specificity*: the proportion of individuals without epilepsy who are correctly identified as negative by the index test.
- *Positive predictive value (PPV)*: probability that individuals who screen positive truly have epilepsy
- *Negative predictive value (NPV)*: probability that individuals who screen negative truly do not have epilepsy

#### Secondary outcomes

- Availability of tools used in countries in SSA
- Psychometric characteristics or properties of tools including ease of use, preference, how it is understood by the population and health professionals, context relevance, appropriateness and feasibility and adaptability (these are often influenced by cultural and individual factors, also the context in which the questionnaire was originally developed.^38^

### Search strategy

Relevant databases, non-database sources and grey literature will be searched for reports on screening tools for detecting epilepsy. PubMed, Embase, Web of Science, LILACS, CINAHL, PsycINFO and Google Scholar, from inception to 31 May 2026, will be searched for studies on screening tools (questionnaires) administered by non-expert physicians in primary care facilities or by non-physicians, including researchers, with no language restrictions. Search terms will include screening tool, screening questions, screening test, screening instruments, diagnostic tool, diagnostic accuracy, epilepsy, sensitivity, specificity, true positive, false positive, negative and positive values, sub-Saharan Africa and SSA, with all applicable synonyms, alternatives, abbreviations, both American and British spellings, as well as plural and singular terms (table 1). We will run additional searches across Cochrane databases, African Journals Online, African Index Medicus, HINARI, Preprint and Thesis repositories. Reference lists of potentially relevant studies will be reviewed, and experts will be contacted to identify additional studies that may have been missed. The search dates, search terms and screening process will be documented clearly to ensure reproducibility.

**Table 1:** Search strategy for PubMed*

Search	Query	Results
#1	((((((((((((((((((((((((Epilepsy*(Title/Abstract)) OR (Convulsion*(Title/Abstract))) OR (“Epileptic syndrome”(Title/Abstract))) OR (“Epileptic disorder”(Title/Abstract))) OR (“Seizure disorder”(Title/Abstract))) OR (“Epileptic condition”(Title/Abstract))) OR (“Seizure episode”(Title/Abstract))) OR (“Seizure activity”(Title/Abstract))) OR (“Seizure attack”(Title/Abstract))) OR (“Epileptic episode”(Title/Abstract))) OR (“Epileptic attack”(Title/Abstract))) OR (“Neurological disorder”(Title/Abstract))) OR (“Falling sickness”(Title/Abstract))) OR (“Recurrent seizures”(Title/Abstract))) OR (“Brain disorder”(Title/Abstract))) OR (“Focal epilepsy”(Title/Abstract))) OR (“Idiopathic epilepsy”(Title/Abstract))) OR (“Generalized seizure disorder”(Title/Abstract))) OR (“Partial seizure disorder”(Title/Abstract))) OR (“Epileptic encephalopathy”(Title/Abstract))) OR (“Refractory epilepsy”(Title/Abstract))) OR (“Symptomatic epilepsy”(Title/Abstract))) OR (“Tonic-clonic epilepsy”(Title/Abstract))) OR (“Fits disorder”(Title/Abstract))) OR (“Absence epilepsy”(Title/Abstract))) OR (“Juvenile myoclonic epilepsy”(Title/Abstract))) OR (“Temporal lobe epilepsy”(Title/Abstract))) OR (“Cerebral convulsive disorder”(Title/Abstract))))	
#2	Search: “True positives”(Title/Abstract)OR “False positives”(Title/Abstract)OR “True negatives”(Title/Abstract)OR “False negatives”(Title/Abstract)OR “ROC curve”(Title/Abstract)OR “AUC”(Title/Abstract)OR “Area under curve”(Title/Abstract)OR “False discovery rate”(Title/Abstract)OR “False omission rate”(Title/Abstract)	
# 3	Search: (#1) AND (#2)	
#4	Search: “Diagnostic accuracy”(Title/Abstract)OR “Screening questionnaires”(Title/Abstract)OR “Screening tools”(Title/Abstract)OR “Screening tests”(Title/Abstract)OR “Diagnostic tools”(Title/Abstract)OR “Diagnostic questionnaires”(Title/Abstract)OR “Screening instruments”(Title/Abstract)OR “Sensitivity”(Title/Abstract)OR “Specificity”(Title/Abstract)OR “Predictive values”(Title/Abstract)OR “Reliability”(Title/Abstract)OR “Validity”(Title/Abstract)	
#5	Search: (#3) AND (#4)	
#6	Search: ((“sub-Saharan Africa”(Title/Abstract)OR SSA(Title/Abstract)OR Africa(Title/Abstract)OR Angola(Title/Abstract)OR Benin(Title/Abstract)OR Botswana(Title/Abstract)OR “Burkina Faso”(Title/Abstract)OR Burundi(Title/Abstract)OR Cameroon(Title/Abstract)OR “Cape Verde”(Title/Abstract)OR “Central African Republic”(Title/Abstract)OR Chad(Title/Abstract)OR Comoros(Title/Abstract)OR “Congo (Brazzaville)“(Title/Abstract)OR “Democratic Republic of the Congo”(Title/Abstract)OR “Côte d'Ivoire”(Title/Abstract)OR Djibouti(Title/Abstract)OR “Equatorial Guinea”(Title/Abstract)OR Eritrea(Title/Abstract)OR Ethiopia(Title/Abstract)OR Gabon(Title/Abstract)OR Gambia(Title/Abstract)OR Ghana(Title/Abstract)OR Guinea(Title/Abstract)OR “Guinea-Bissau”(Title/Abstract)OR Kenya(Title/Abstract)OR Lesotho(Title/Abstract)OR Liberia(Title/Abstract)OR Madagascar(Title/Abstract)OR Malawi(Title/Abstract)OR Mali(Title/Abstract)OR Mauritania(Title/Abstract)OR Mauritius(Title/Abstract)OR Mozambique(Title/Abstract)OR Namibia(Title/Abstract)OR Niger(Title/Abstract)OR Nigeria(Title/Abstract)OR Rwanda(Title/Abstract)OR “Sao Tome and Principe”(Title/Abstract)OR Senegal(Title/Abstract)OR Seychelles(Title/Abstract)OR “Sierra Leone”(Title/Abstract)OR Somalia(Title/Abstract)OR “South Africa”(Title/Abstract)OR Sudan(Title/Abstract)OR Eswatini(Title/Abstract)OR Tanzania(Title/Abstract)OR Togo(Title/Abstract)OR Uganda(Title/Abstract)OR Zambia(Title/Abstract)OR Zimbabwe(Title/Abstract)))	
#7	(#5) AND (#6)	

* The search strategy will be adapted as appropriate for other databases. All searches will be re-run just before the final analyses and any further eligible studies identified will be included.

### Study selection

The search output will be managed, collated and deduplicated using EndNote.^44^ The deduplicated articles will be exported to Rayyan^45^ for screening and selection. Two reviewers (EKD and AD-A) will independently screen the titles and abstracts of identified studies using a piloted eligibility flowchart (online supplemental file 3), developed from the patient/population, index test(s) and target condition. The full texts of potentially relevant studies will be further reviewed, and those meeting the prespecified eligibility criteria for inclusion will be included. The PRISMA flow diagram^39^ will be used to document the flow of studies and reasons for exclusion. Discrepancies will be resolved through consensus.

### Data extraction and management

Two reviewers (EKD and AD-A) will independently extract data from each included study using a pre-tested data extraction tool designed for this purpose and resolve disagreements through discussion. The information to be extracted from each study will include the following: country of the study, language of the screening questionnaire, source and number of persons with epilepsy and without epilepsy, target condition and its definition, index test, reference standard test, definition of a positive screen, setting of the interview and characteristics of the interviewer. Data on the psychometric characteristics or properties of the tools will also be extracted, including feasibility, adaptability, expertise required to deliver the tool, ease of use, patient preference and cost of application, if available. If available, data on true negatives, false negatives, true positives and false positives will be extracted from each study to complete a 2×2 contingency table and estimate the pooled sensitivity, specificity and DOR. Studies without explicit 2×2 data will not be included in the meta-analysis unless true positives, false positives, true negatives and false negatives can be calculated from reported metrics. Cases in which 2×2 data are not explicitly reported will be addressed using the reported metrics to derive the necessary data where possible. For example, if studies report sensitivity, specificity or other relevant metrics such as DORs, standard statistical formulas will be used to calculate and populate the 2×2. If these calculations cannot be performed due to missing or incomplete information, this limitation will be documented and clearly explained. The primary study authors will be contacted regarding missing or unclear data. Any disagreements will be resolved by consensus.

### Risk of bias (quality) assessment

The quality of included studies will be independently assessed by two reviewers for risk of bias using diagnostic accuracy QUADAS-2^46 47^ (online supplemental file 4). This tool is based on a series of signalling questions across four risk-of-bias domains: (1) patient selection, (2) index test(s), (3) reference standard and (4) flow and timing. Responses to each of the signalling questions are ‘yes’, ‘no’ and ‘unclear’. Each domain in the QUADAS-2 tool will be rated as ‘low risk’, ‘high risk’ or ‘unclear risk’ of bias. Studies with a high risk of bias in multiple domains may be excluded from the meta-analysis or subjected to sensitivity analysis to assess the impact of potential biases on the results. An overall judgement regarding the risk of bias for each study will be made based on the individual domain assessments. Any reviewer discrepancies will be resolved through discussion or consulting a third reviewer.

### Strategy for data synthesis

The Wilson method will be used to calculate 95% CIs for each diagnostic accuracy parameter.^48^ The DOR, which is the ratio of the proportion that tests positive among those with the target condition compared with the proportion that tests positive among those without the target condition, will be calculated as follows: [sensitivity/ (1 − sensitivity)/ (1 − specificity)/specificity]. The DOR has the advantage of being independent of disease prevalence. A diagnostic test will be considered discriminative if its DOR is >1.^49^ PPV and NPV will be calculated using the reported sensitivity and specificity estimates for each study. Because predictive values are strongly influenced by disease prevalence, PPV and NPV will be estimated across a plausible prevalence range of ≥0.1% to reflect variability in epilepsy prevalence across SSA, using these formulas:

PPV= [(sensitivity) (prevalence)/((sensitivity) (prevalence) + (1 – specificity) (1 – prevalence)

NPV = (specificity) (1 – prevalence)/ (specificity) (1 – prevalence) + (1 – sensitivity) (prevalence)].

A meta-analysis will be conducted using a bivariate random-effects model to generate summary estimates of sensitivity, specificity and DOR per person. To account for the correlation between sensitivity and specificity, the bivariate model will be used as the primary method. This model simultaneously estimates both sensitivity and specificity for each study while accounting for their inherent correlation. In cases where test thresholds are highly variable or studies report multiple thresholds, the hierarchical summary ROC model may be considered. This model extends the bivariate approach by allowing for threshold variation and estimating overall diagnostic performance. A summary ROC curve will be plotted to assess the overall diagnostic performance of the index tests.^28^ The closer the curve approaches the upper-left corner, the higher the overall performance.^50^ A perfect test has an AUC of 1. The AUC-ROC will be estimated using sensitivity and specificity values across different threshold settings. Data from the included studies will be used to generate ROC curves and compute the AUC. Summary estimates, including pooled sensitivity, specificity and DOR, will be generated with associated 95% CIs; p<0.05 will be considered statistically significant. To remove the need to adjust for confounders, the analysis will be restricted to studies with head-to-head comparisons in which the index and reference tests were performed on the same participants. Given the geographical context of this systematic review covering 49 countries in SSA, we do not anticipate sparse data that will affect head-to-head comparison. Nevertheless, if data are sparse, we will report narratively.

### Heterogeneity assessment and subgroup analysis

Heterogeneity, arising from variations in study design, participant characteristics or outcomes between or within studies, will be investigated graphically and statistically. The I² statistic, which describes the percentage of variability due to heterogeneity rather than chance,^51^ will be estimated. The four levels will classify the I² statistic: 0%–24% as unimportant, 25%–49% as moderate, 50%–74% as substantial and 75%–100% as considerable heterogeneity.^51^ Cochrane’s Q statistics and Tau^52^ will also be presented, and p<0.05 was the cut-off for statistically significant heterogeneity.

Subgroup analysis will be conducted to explore potential sources of heterogeneity in the diagnostic accuracy of epilepsy screening tools across different settings in SSA. Key factors for subgroup analysis may include the test setting (rural vs urban) and population characteristics such as age, sex and the prevalence of epilepsy in the study region. Additional subgroup analyses based on tool type, mode of administration (telephone vs in-person), administrator type and epilepsy subtype (convulsive vs non-convulsive). Regarding the administration method, subgroups will be based on whether the test is performed in a clinic or primary care setting, at home, in person or by telephone. Additionally, for epilepsy subtypes, subgroups will include different forms of epilepsy (eg, convulsive vs non-convulsive) to examine potential variations in screening accuracy across these categories. On country-level differences, variation will be assessed through subgroup analyses and meta-regression, incorporating country or region as a covariate. Subgroup analyses will be conducted only when at least five studies are available within a subgroup to ensure stability of parameter estimates in diagnostic test accuracy models. Subgroups with fewer than five studies will be summarised narratively without formal statistical pooling. All statistical analyses will be performed in Stata, applying the metandi command for diagnostic test accuracy meta-analysis.

### Assessment of the certainty of evidence using the GRADE approach

The overall evidence of the systematic review will be graded using the Grading of Recommendations Assessment, Development and Evaluation (GRADE) approach. The GRADE approach assesses the following domains: (1) risk of bias (systematic errors in study design, conduct or analysis that distort results, leading to incorrect conclusions about exposure-outcome links); (2) imprecision (random error in study results often due to chance resulting from small sample sizes or measurement variability, leading to wide CIs and uncertainty about the true effect); (3) inconsistency (disagreement between different estimates and sources of evidence in which results from included studies in a systematic review vary significantly and unpredictably, reducing confidence in the overall findings); (4) indirectness (when the evidence does not perfectly match the review’s specific question, leading to lower confidence in the findings and occurring due to differences in the review’s participants, interventions/exposure, comparisons employed and the outcomes being measured; and (5) publication bias (the tendency for studies for statistically significant or positive results to be published more often, faster and in more prominent journals than studies with ‘negative’, null or inconclusive findings, creating a distorted view of the evidence). The certainty of evidence will be graded as high, moderate, low or very low. The certainty of the evidence for each outcome will be summarised in a table detailing the study design, number of studies and participants and corresponding relative and absolute effect estimates.^53^ High-quality evidence implies that further research is very unlikely to change confidence in the estimate of effect, whereas very low-quality evidence indicates that the estimate of effect is very uncertain.^54^

### Integration of risk of bias into GRADE assessment

In this review, the QUADAS-2 framework will be used to evaluate the risk of bias in each diagnostic accuracy study included in the review. The risk of bias will be assessed across four domains: patient selection, index test, reference standard and flow and timing. Each study will be categorised as having low, high or unclear risk of bias for each domain. As part of the GRADE framework, the results of the QUADAS-2 risk-of-bias assessment will be integrated into the overall evaluation of evidence quality. Specifically, the risk-of-bias ratings from QUADAS-2 will inform the GRADE domains of risk of bias, indirectness, inconsistency, imprecision and publication bias. Any identified risk of bias will be considered when determining confidence in the diagnostic test accuracy, and adjustments will be made to the GRADE rating accordingly.

## Ethics and dissemination

Ethical approval is not required for a systematic review, as it collates publicly available data. The results will be presented to health professionals, support groups, policymakers, researchers and non-governmental organisations. It will be disseminated through conferences and peer-review publications. This protocol has been registered on the International Prospective Register of Systematic Reviews (PROSPERO) with registration ID CRD42024566976.

## Discussion

This systematic review and meta-analysis aims to provide a comprehensive evaluation of the accuracy of low-cost, easy-to-use and context-sensitive screening questionnaires to identify individuals with epilepsy in community and primary care settings across SSA. Despite the significant burden of epilepsy in this region, access to diagnostic tools and neurologists remains limited, especially in rural and resource-constrained areas.^7 55 56^ Screening questionnaires play a crucial role in the early detection and management of epilepsy, potentially enabling early intervention and ultimately reducing associated morbidity and mortality.^18 57 58^

We aim to identify screening tools and synthesise evidence to shed light on their performance in terms of validation across diverse settings, cultures, socio-demographic circumstances and population subgroups. Understanding these accuracy metrics will not only highlight the strengths and weaknesses of current screening tools and criteria but also provide insight into their adaptability and reliability across varied socio-economic and cultural contexts in SSA. This analysis will also help identify significant gaps in the effectiveness of these tools, which could inform the development or refinement of more culturally and contextually appropriate screening methods.

Screening performance is likely to be influenced in the community more than in clinic settings, with communities likely to register lower performance given that non-convulsive seizures may be less recognised, understood or interpreted by lay respondents. Screening performance in paediatric populations may be affected more because of possible misclassification of non-epileptic events such as febrile seizures, and many others may lead to an increase in false positives, thereby reducing the specificity and overall performance. We also anticipate that validated questionnaires will perform better overall than locally adapted or unvalidated tools. By synthesising data from multiple studies, this review will support policymakers, healthcare providers and researchers in making evidence-based decisions regarding the adoption or modification of specific screening tools. This could ultimately lead to more effective and equitable identification of epilepsy, particularly among underserved and marginalised populations in SSA, contributing to the global efforts to address the treatment gap for epilepsy in low-income and middle-income countries.

### Implications of the anticipated review findings

Identifying the strengths and weaknesses of current screening tools will help adapt more accurate and affordable questionnaires suited to the realities of SSA. Strengthening these methods can reduce the burden of epilepsy and provide policymakers with evidence to prioritise screening programmes. Highlighting the existing gaps may also stimulate future research into more effective and culturally appropriate screening approaches.

## Supplementary material

10.1136/bmjopen-2026-116684 online supplemental file 1

10.1136/bmjopen-2026-116684 online supplemental file 2

10.1136/bmjopen-2026-116684 online supplemental file 3

10.1136/bmjopen-2026-116684 online supplemental file 4
