# Supplementary material for: Accuracy of epilepsy screening tools in community and primary care settings across countries in Sub-Saharan Africa: systematic review and meta-analysis protocol
Source: BMJ Open. 2026 May 7;16(5):e116684. doi: 10.1136/bmjopen-2026-116684 (PMC13157775; doi:10.1136/bmjopen-2026-116684)
Supplement: online supplemental file 2 [file bmjopen-16-5-s002.pdf]

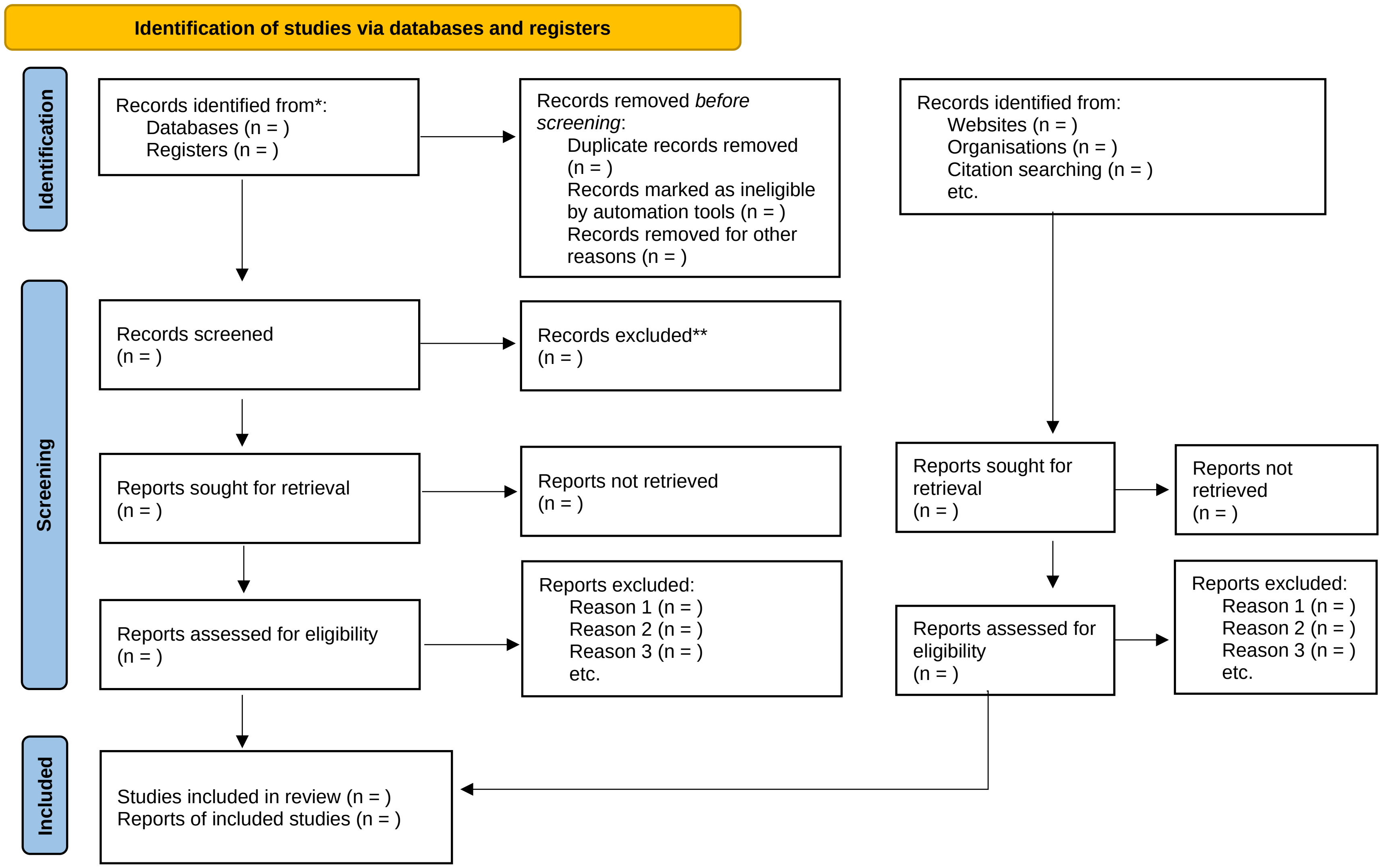

SUPLEMENTAL FILE 3 PRISMA-P 2020 Flow Diagram to show studies retrieved from electronic databases and other sources for inclusion and flow to the final stage with studies included in the systematic review
