# Supplementary figures and images for: Accuracy of epilepsy screening tools in community and primary care settings across countries in Sub-Saharan Africa: systematic review and meta-analysis protocol
Source: BMJ Open. 2026 May 7;16(5):e116684. doi: 10.1136/bmjopen-2026-116684 (PMC13157775; doi:10.1136/bmjopen-2026-116684)

Figure 1: Study selection flow chart

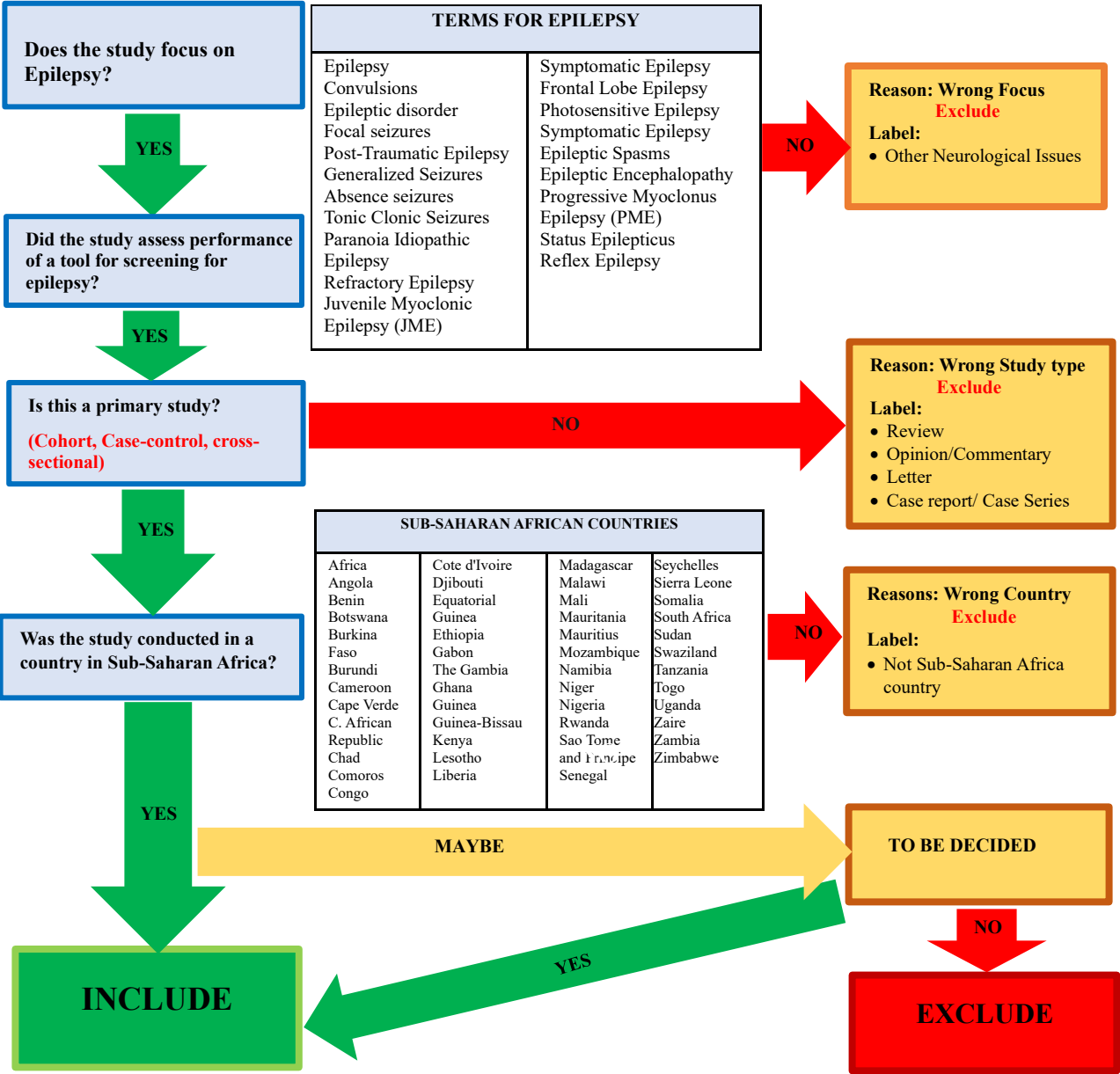

Supplement: online supplemental file 3 [file bmjopen-16-5-s003.pdf]
