# Supplementary material for: Accuracy of epilepsy screening tools in community and primary care settings across countries in Sub-Saharan Africa: systematic review and meta-analysis protocol
Source: BMJ Open. 2026 May 7;16(5):e116684. doi: 10.1136/bmjopen-2026-116684 (PMC13157775; doi:10.1136/bmjopen-2026-116684)
Supplement: online supplemental file 4 [file bmjopen-16-5-s004.pdf]

Supplementary 2 Table QUADAS-2 tool (Quality Assessment of Diagnostic Accuracy Studies): Risk of bias and applicability judgments

**Domain 1: Patient selection**	
**A. Risk of bias**	
**Describe methods of patient selection:**	
• **Was a consecutive or random sample of patients enrolled?**	Yes/No/Unclear
• **Was a case-control design avoided?**	Yes/No/Unclear
• **Did the study avoid inappropriate exclusions?**	Yes/No/Unclear
**Could the selection of patients have introduced bias?**	RISK: LOW/HIGH/UNCLEAR
**B. Concerns regarding applicability**	
**Describe included patients (prior testing, presentation, intended use of index test and setting):**	
**Is there concern that the included patients do not match the review question?**	CONCERN: LOW/HIGH/UNCLEAR
**Domain 2: Index test(s)** ***(if more than 1 index test was used, please complete for each test)***	
**A. Risk of bias**	
**Describe the index test and how it was conducted and interpreted:**	
• **Were the index test results interpreted without knowledge of the results of the reference standard?**	Yes/No/Unclear
• **If a threshold was used, was it pre-specified?**	Yes/No/Unclear
**Could the conduct or interpretation of the index test have introduced bias?**	RISK: LOW/HIGH/UNCLEAR
**B. Concerns regarding applicability**	
**Is there concern that the index test, its conduct, or interpretation differ from the review question?**	CONCERN: LOW/HIGH/UNCLEAR
**Domain 3: Reference standard**	
**A. Risk of bias**	
**Describe the reference standard and how it was conducted and interpreted:**	
• **Is the reference standard likely to correctly classify the target condition?**	Yes/No/Unclear
• **Were the reference standard results interpreted without**	Yes/No/Unclear

**knowledge of the results of the index test?**	
**Could the reference standard, its conduct, or its interpretation have introduced bias?**	RISK: LOW/HIGH/UNCLEAR
**B. Concerns regarding applicability**	
**Is there concern that the target condition as defined by the reference standard does not match the review question?**	CONCERN: LOW/HIGH/UNCLEAR
**Domain 4: Flow and timing**	
**A. Risk of bias**	
**Describe any patients who did not receive the index test(s) and/or reference standard or who were excluded from the 2x2 table (refer to flow diagram):**<br><br>**Describe the time interval and any interventions between index test(s) and reference standard:**	
• **Was there an appropriate interval between index test(s) and reference standard?**	Yes/No/Unclear
• **Did all patients receive a reference standard?**	Yes/No/Unclear
• **Did patients receive the same reference standard?**	Yes/No/Unclear
• **Were all patients included in the analysis?**	Yes/No/Unclear
**Could the patient flow have introduced bias?**	RISK: LOW/HIGH/UNCLEAR
