# Supplementary material for: Accuracy of epilepsy screening tools in community and primary care settings across countries in Sub-Saharan Africa: systematic review and meta-analysis protocol
Source: BMJ Open. 2026 May 7;16(5):e116684. doi: 10.1136/bmjopen-2026-116684 (PMC13157775; doi:10.1136/bmjopen-2026-116684)
Supplement: Supplementary file 5 [file bmjopen-2026-116684.reviewer_comments.pdf]

PEER REVIEW HISTORY

BMJ Open publishes all reviews undertaken for accepted manuscripts. Reviewers are asked to complete a checklist review form and are provided with free text boxes to elaborate on their assessment. These free text comments are reproduced below.

ARTICLE DETAILS

Title (Provisional)

Accuracy of Epilepsy Screening Tools in community and primary care settings across Countries in sub-Saharan Africa: Systematic Review and Meta-Analysis Protocol

Authors

Darkwa, Emmanuel Kwame; Cross, Helen; Adjei, Patrick; Newton, Charles RJC; Sen, Arjune; Akpalu, Albert; Sander, J W; Danso-Appiah , Anthony

---

VERSION 1 - REVIEW

**Reviewer**	**1**
**Name**	**Lemoine, Émile**
**Affiliation**	**Centre Hospitalier de l'Université de Montréal**
**Date**	**16-Feb-2026**
**COI**	**None**

This study presents a protocol for a Systematic review of diagnostic test accuracy (DTA) evaluating the performance of screening questionnaires for the diagnosis of epilepys in SSA countries. The research question is important and the methodology is well defined and in accordance with PRISMA-DTA and Cochrane recommendations. Still, the manuscript would benefit from major changes to improve the readability and strenghten the methodology.

As a general comment, the manuscript is too long with redundant sections. The abstract exceeds typical limits (4-page long). Most of the Rationale section is redunant with the Introduction. The review questions and objectives are nearly identical and the SDG paragraph adds little. From the Reference standard section onward, the bullet point format decreases readability with some sentences being incomplete (e.g.: p. 12, l. 286; p.22, l. 501); this should be converted to prose. Also, the Definitions section may be unnecessary as it describes basic concepts that most readers will know; it could be moved to an appendix.

The methodological issues are detailed below. More importantly, the reference standard is imprecise, over-emphasizes EEG findings, and is not consistent with the Internal League Against Epilepsy's definition of epilepsy (Fisher et al., 2014).

1. The introduction adequately frames the burden of epilepsy but should preview what screening tools exist and why their accuracy might vary specifically in SSA contexts. Providing brief accuracy estimates from prior validation studies would orient readers and justify the review.

2. The two review questions ask essentially the same thing: diagnostic accuracy, then variation in accuracy. Consolidate them or sharpen the distinction: for example, Question 1 addresses pooled accuracy; Question 2 addresses sources of heterogeneity (including inter-test performance variability). The objectives mirror the questions and add nothing; consider deleting or differentiating them.

3. The exclusion of studies administered by epilepsy specialists requires justification (many validation studies use specialists to establish criterion validity). Clarify whether you are excluding studies where specialists administered the screening tool versus those where specialists served as the reference standard. Consider including specialist-administered studies and examining administrator type as a subgroup or sensitivity analysis.

3. Studies reporting aggregated multi-country performance data should be explicitly addressed and not excluded. Also, how will country-level heterogeneity be handled?

4. The listed index tests (Kilifi, WHO, Zambian 5-item) are examples. Authors should state explicitly whether any screening questionnaire is eligible or only named, previously validated instruments.

5. "Availability of screening tools" is listed as a primary outcome but is a descriptive finding unrelated to diagnostic accuracy. It should be removed from primary outcomes or reframe it as contextual information. Coincidentally, PPV and NPV arguably belong to the primary outcome along with sensitivity and specificity.

6. The epilepsy definition should conform with the current ILAE practical clinical definition (Fisher et al., 2014). Other terminology should follow ILAE standards: use "focal impaired consciousness seizure" rather than "impaired awareness". The example of atonic seizures under "non-convulsive epilepsy" is inappropriate; atonic seizures are a generalized seizure type, not a form of non-convulsive epilepsy.

7. The data extraction section states that data will be "independently verified," but the correct process is "independently extracted" by two reviewers who are blinded to each other's results, followed by consensus. Revise the wording accordingly.

8. It is unclear why latent class analysis is used. This method applies when no adequate reference standard exists, yet the protocol specifies neurologist diagnosis and EEG as references. Explain under what circumstances latent class analysis would be used, or remove it.

9. The assumption of 1% prevalence for calculating predictive values needs justification. Epilepsy prevalence in SSA community surveys ranges from 0.5% to over 3%. The authors could present predictive values across a plausible prevalence range rather than a single point estimate.

10. State the statistical software to be used (e.g., R with mada, Stata with metandi).

11. The subgroup analyses are appropriate, but the methodology should pre-specify the minimum number of studies required per subgroup. Also, the discussion could benefit from stating an expected direction of effect for each subgroup to demonstrates a priori hypotheses.

12. It appears that one of the main justification for the review is comparing screening tools head-to-head. Describe how this comparison will be conducted (e.g., network meta-analysis, indirect comparison, or qualitative assessment if data are sparse).

13. Publication bias assessment is mentioned under the GRADE framework but the methodology for this analysis is not specified.

14. A clear timeline with projected dates should be included to clarify which steps are ongoing or planned upon publication of the protocol.

15. The discussion should include references and formulate hypotheses on the projected findings

---

VERSION 1 - AUTHOR RESPONSE

Reviewer #1

General comments

Comment: This study presents a protocol for a Systematic review of diagnostic test accuracy (DTA) evaluating the performance of screening questionnaires for the diagnosis of epilepsy in SSA countries. The research question is important, and the methodology is well defined and in accordance with PRISMA-DTA and Cochrane recommendations. Still, the manuscript would benefit from major changes to improve the readability and strengthen the methodology.

Response: *We thank the reviewer for the careful assessment of our manuscript and for the constructive suggestions. We appreciate the positive comments regarding the relevance of the research question and the methodological alignment with PRISMA-DTA and Cochrane recommendations. We have revised the manuscript as appropriate to improve clarity, readability, and structure.*

Comment: As a general comment, the manuscript is too long with redundant sections. The abstract exceeds typical limits (4-page long).

Response: *Thank you, we have shortened the manuscript by removing the full section on 'Expected outcomes and redundant sentences.*

Comment: Most of the Rationale section is redundant with the Introduction.

Response: *Thank you. We have carefully reviewed the rationale section and removed redundant*

statements and overlapping sentences with the Introduction.

Comment: The review questions and objectives are nearly identical.

Response: *We have revised the review questions to introduce clarity.*

Comment: The SDG paragraph adds little.

Response: *Thank you for the comment. Whilst we recognize the reviewer's concern, we consider the reference to the Sustainable Development Goals (SDGs) important for contextualizing the relevance of epilepsy and epilepsy care in SSA, particularly in relation to health equity and access to diagnosis and treatment in resource-limited settings.*

Comment: From the Reference standard section onward, the bullet point format decreases readability and some sentences are incomplete. This should be converted to prose.

Response: *We have changed the bullet-point format to improve readability and flow and moved the whole to the section before the Methods (Page 9, lines 241-330). In addition, incomplete sentences have been corrected.*

Comment: The Definitions section may be unnecessary as it describes basic concepts that most readers will know; it could be moved to an appendix.

Response: *We appreciate this suggestion. We have moved the definitions section from the methods to the section immediately following the rationale, where it now serves to clarify key terms used in the review. We believe this placement helps orient readers who may not be familiar with specific terminology used in diagnostic accuracy studies of epilepsy screening tools while avoiding disruption of the methodological flow.*

Specific comments

Comment 1: The introduction adequately frames the burden of epilepsy but should preview what screening tools exist and why their accuracy might vary specifically in SSA contexts. Providing brief accuracy estimates from prior validation studies would orient readers and justify the review.

Response: *Thank you for this important comment. We have now added a preview of commonly used epilepsy screening questionnaires in sub-Saharan Africa and highlighted the variation and determinants of accuracy such as cultural, stigmatization, linguistic and epidemiological factors as well as how symptoms are understood, interpreted and reported. We have added the preview below to the introduction (Pages 6-7, line 162-177).*

"Community and primary care screening for epilepsy in resource-limited settings commonly

use structured questionnaires,[1] of which several have been applied in SSA, including the Kilifi epilepsy questionnaire,[2] WHO screening questions,[3] and the Zambian five-item questionnaire.[4] These questionnaires with varying accuracies (sensitivities ranging from 60% to over 95% and specificities 50% to over 90%),[2 4 5] are usually administered by non-specialist or community health workers as a first step towards the identification of suspected epilepsy cases. For example, a community-based study conducted in rural Kenya reported sensitivity of 48.6% and specificity of 100%,[6] and another from rural Zambia reported sensitivity of 64% and specificity of 86%.[4] Linguistic, cultural, questionnaire translation and seizures description diversity, as well as how symptoms are understood, interpreted and reported, are the common factors influencing screening accuracy in SSA.[2 4-6] Currently, no single universally accepted screening instrument exists in SSA, and given that performance appears to be context-sensitive, comprehensive synthesis of data to determine screening accuracy of available tools is warranted to help guide the selection, adaptation, and utility of screening questionnaires in community and primary care settings across countries in SSA."

1. Placencia M, Sander J, Shorvon S, Ellison R, Cascante S. Validation of a screening questionnaire for the detection of epileptic seizures in epidemiological studies. Brain. 1992;115(3):783-94.
2. Mung'ala-Odera V, Meehan R, Njuguna P, Mturi N, Alcock K, Carter J, et al. Validity and reliability of the 'Ten Questions' questionnaire for detecting moderate to severe neurological impairment in children aged 6–9 years in rural Kenya. Neuroepidemiology. 2004;23(1-2):67-72.
3. Preux P-M, Druet-Cabanac M. Epidemiology and aetiology of epilepsy in sub-Saharan Africa. The Lancet Neurology. 2005;4(1):21-31.
4. Birbeck GL, Kalichi EM. Epilepsy prevalence in rural Zambia: a door-to-door survey. Tropical Medicine & International Health. 2004;9(1):92-5.
5. Watila MM, Balarabe SA, Komolafe M, Igwe SC, Fawale MB, van Diessen E, et al. Translation and validation of an epilepsy-screening questionnaire in three Nigerian languages. Epilepsy & Behavior. 2021;114:107604.
6. Ngugi AK, Bottomley C, Chengo E, Kombe MZ, Kazungu M, Bauni E, et al. The validation of a three-stage screening methodology for detecting active convulsive epilepsy in population-based studies in health and demographic surveillance systems. Emerging themes in epidemiology. 2012;9:1-8.

Comment 2: The two review questions ask essentially the same thing: diagnostic accuracy, then variation in accuracy. Consolidate them or sharpen the distinction: for example, Question 1 addresses pooled accuracy; Question 2 addresses sources of heterogeneity (including inter-test performance variability). The objectives mirror the questions and add nothing; consider deleting or differentiating them.

Response: *Thank you. The review questions and objectives have been revised to introduce clarity by differentiating between estimating pooled diagnostic accuracy (question 1) and assessing factors associated with variability in accuracy across studies (question 2). We have deleted the objectives (Page 8, lines 224-234).*

Comment 3: The exclusion of studies administered by epilepsy specialists requires justification (many validation studies use specialists to establish criterion validity). Clarify whether you are excluding studies where specialists administered the screening tool versus those where specialists served as the reference standard. Consider including specialist-administered studies and examining administrator type as a subgroup or sensitivity analysis.

Response: *Thank you for this important comment. After careful consideration and given that many validation studies use specialists to establish criterion validity, studies administered by epilepsy specialists will be included (Page 13, lines 362-364).*

Comment 3: Studies reporting aggregated multi-country performance data should be explicitly addressed and not excluded. Also, how will country-level heterogeneity be handled?

Response: *Thank you. When country-specific diagnostic accuracy data are provided, they will be extracted separately and considered as independent estimates. If only aggregated data are available and there is no way of disaggregating, the study will be included as a single multi-country dataset and interpreted accordingly. We have updated the criteria for considering studies for inclusion in this review to include* studies reporting aggregated multi-country performance data *(Page 13, lines 360-362).*

On country-level differences, we will assess variation through subgroup analyses and meta-regression, incorporating country or region as a covariate where data are available (Page 23, lines 644-645).

Comment 4: The listed index tests (Kilifi, WHO, Zambian 5-item) are examples. Authors should state explicitly whether any screening questionnaire is eligible or only named, previously validated instruments.

Response: *The listed index tests are examples of available screening questionnaires in SSA. This systematic review aims to include all existing epilepsy screening questionnaires for identifying suspected cases and not necessarily those that have been validated. The eligibility criteria have been updated to clarify any confusion and to explicitly state that any screening questionnaire designed to detect epilepsy in community or primary care settings will be eligible for inclusion regardless of validation status (Page 13, lines 378-380).*

Comment 5: Availability of screening tools" is listed as a primary outcome but is a descriptive finding unrelated to diagnostic accuracy. It should be removed from primary outcomes or reframe it as contextual information. Coincidentally, PPV and NPV arguably belong to the primary outcome along with sensitivity and specificity.

Response: *Thank you, the identification of existing tools across countries in SSA is key in this systematic review as there is paucity of data on existing tools. We believe exploring existing screening tools in this region is a first step towards assessing tool-specific performance in order to make recommendations for questionnaires that combine adaptability, ease of administration and interpretation, not requiring specialist skills, among others, for resource-limited countries across SSA. The review outcomes have been reorganized to make sensitivity, specificity, PPV, and NPV as our primary outcomes (Page 14, lines 405-422).*

Comment 6: The epilepsy definition should conform with the current ILAE practical clinical definition (Fisher et al., 2014). Other terminology should follow ILAE standards: use "focal impaired consciousness seizure" rather than "impaired awareness". The example of atonic seizures under "non-convulsive epilepsy" is inappropriate; atonic seizures are a generalized seizure type, not a form of non-convulsive epilepsy.

Response: *Thank you. We have updated the manuscript to reflect the ILAE's definition of epilepsy (Fisher et al., 2014). All other terminologies now follow the ILAE standards including the adoption of the term "focal impaired consciousness seizure" (Pages 9, lines 241-257).*

Comment 7: The data extraction section states that data will be "independently verified," but the correct process is "independently extracted" by two reviewers who are blinded to each other's results, followed by consensus. Revise the wording accordingly.

Response: *Thank you. The wording has been revised to read "Two reviewers will independently extract data and resolve disagreements by consensus (Page 20, lines 555-557).*

Comment 8: It is unclear why latent class analysis is used. This method applies when no adequate reference standard exists, yet the protocol specifies neurologist diagnosis and EEG as references. Explain under what circumstances latent class analysis would be used,or remove it.

Response: *Thank you, we have removed the section on latent class analysis as the specified reference standard (clinical diagnosis by a neurologist) is reliable (Page 23, lines 650-653).*

Comment 9: The assumption of 1% prevalence for calculating predictive values needs justification. Epilepsy prevalence in SSA community surveys ranges from 0.5% to over 3%. The authors could present predictive values across a plausible prevalence range rather than a single point estimate.

Response: *We agree predictive values are heavily influenced by disease prevalence, and relying on a fixed prevalence of 1% may not capture the variability across sub-Saharan African settings. We have updated the manuscript to estimate positive and negative predictive values over a realistic prevalence range of ≥ 0.1%. (Page 21, lines 595-599).*

Comment 10: State the statistical software to be used (e.g., R with mada, Stata with metandi).

Response: *All statistical analyses will be performed using Stata with the metandi command for diagnostic test accuracy meta-analysis. We have updated the manuscript to reflect (Pages 23, lines 648-650).*

Comment 11: The subgroup analyses are appropriate, but the methodology should pre-specify the minimum number of studies required per subgroup. Also, the discussion could benefit from stating an expected direction of effect for each subgroup to demonstrates a priori hypotheses.

Response: *Thank you. We have revised the Methods section to explicitly state that at least 5 studies are required before conducting subgroup analyses, to ensure stability of parameter estimation in diagnostic test accuracy models. Subgroups with fewer studies will be summarized narratively without formal statistical pooling. (Page 23, lines 645-648). We have also added few statements in the discussion about priori hypotheses of the expected direction of effect for each subgroup (Pages 25 lines 716-722).*

Comment 12: It appears that one of the main justification for the review is comparing screening tools head-to-head. Describe how this comparison will be conducted (e.g., network meta-analysis, indirect comparison, or qualitative assessment if data are sparse).

Response*: Thank you for the important comment. Given the geographical context of this systematic review covering 49 countries in SSA, we do not anticipate sparse data to affect head-to-head comparison. Nevertheless, if data are sparse, we will report narratively and not conduct indirect or network meta-analysis because of the inherent limitations of these approaches (Page 22, lines 622-624).*

Comment 13: Publication bias assessment is mentioned under the GRADE framework but the methodology for this analysis is not specified.

Response: *Thank you for this important comment. In this review, we will minimize publication bias by conducting broad searches across multiple databases, grey literature, and non-database sources. Given that publication bias can arise when search strategies are not sufficiently comprehensive, we have described comprehensive terms/concepts and search strategy that have been calibrated on sensitivity and specificity to minimize missing relevant studies. Although statistical tests for publication bias, such as Egger's test, can be useful, they are not a substitute for comprehensive searching for eligible studies in a systematic review. In addition, the interpretation of these quantitative assessments should be approached with caution. For example, funnel plot asymmetry may suggest publication bias, but it may also result from heterogeneity or small-study effects. Conversely, symmetry in a funnel plot does not necessarily mean that all relevant studies have been identified. Therefore, within the GRADE framework, publication bias will be assessed holistically. The certainty of evidence will be downgraded where there is reasonable suspicion of publication bias, taking into account the comprehensive nature of the search strategy and the range of sources searched, including both database and non-database sources.*

Comment 14*:* A clear timeline with projected dates should be included to clarify which steps are ongoing or planned upon publication of the protocol.

Response: *Thank you, we have included projected timelines outlining the stages of the review process, including literature search, screening, data extraction, analysis, and manuscript preparation, to clarify the current status and future steps ((Page 12, Line 340-343).*

Comment 15: The discussion should include references and formulate hypotheses on the projected findings

Response: *We have added references in the discussion and outlined anticipated findings. The following statements about expected outcomes have been added to the discussions (Pages 25 lines 716-722).*

"Screening performance is likely to be influenced in the community than clinic settings, with communities likely to register lower performance given that non-convulsive seizures may be less recognized, understood or interpreted by lay respondents. Screening performance in pediatric populations may be affected more due to possible misclassification of non-epileptic events such as febrile seizures and many others may lead to an increase in false positives, thereby reducing the specificity and overall performance. We also anticipate that validated questionnaires will perform better overall than locally adapted or unvalidated tools."

VERSION 2 - REVIEW

**Reviewer**	**1**
**Name**	**Lemoine, Émile**
**Affiliation**	**Centre Hospitalier de l'Université de Montréal**
**Date**	**11-Apr-2026**
**COI**	

The authors have addressed my concerns.

VERSION 2 - AUTHOR RESPONSE

Reviewer 1: "The authors have addressed my concerns."

Response: We thank the reviewer for the positive feedback that all concerns have been addressed.

We have uploaded a clean version of the manuscript and a marked copy with tracked changes
